# Finding missing diversity from synonyms of *Haplopteris* (Pteridaceae)

**DOI:** 10.3897/phytokeys.178.67622

**Published:** 2021-05-27

**Authors:** Zuo-Ying Wei, Zeng-Qiang Xia, Xian-Chun Zhang, Jian-Guo Cao, Yue-Hong Yan

**Affiliations:** 1 Shenzhen key laboratory for Orchid Conservation and Utilization, National Orchid Conservation center of China and the Orchid Conservation & research Center of Shenzhen, Shenzhen 518114, China Shanghai Normal University Shanghai China; 2 College of Life and Environmental Sciences, Shanghai Normal University, Shanghai 201602, China National Orchid Conservation center of China and the Orchid Conservation & research Center of Shenzhen Shenzhen China; 3 CAS Center for Excellence in Molecular Plant Sciences, Shanghai Institute of Plant Physiology and Ecology, Chinese Academy of Sciences, 300 Fenglin Road, Shanghai 200032, China Shanghai Institute of Plant Physiology and Ecology, Chinese Academy of Sciences Shanghai China; 4 State Key Laboratory of Systematic and Evolutionary Botany, Institute of Botany, The Chinese Academy of Sciences, Beijing 100093, China Institute of Botany, The Chinese Academy of Sciences Beijing China

**Keywords:** *Haplopteris*, molecular phylogeny, new combination, nomenclature, Pteridaceae, taxonomy

## Abstract

Although taxonomists target the remote wild regions to discover new species, taxa lacking a comprehensive and modern systematic treatment may be the new hotspot for biodiversity discovery. The development of molecular systematics integrated with microscopic observation techniques has greatly improved the ability of taxonomists to identify species correctly. *Vittariacentrochinensis* Ching ex J.F. Cheng, regarded as a synonym of *Haplopterisfudzinoi* (Makino) E.H.Crane, remained hidden from the eyes of fern taxonomists for more than 20 years. Herein, we collected several population samples of *V.centrochinensis* by performing molecular phylogenetic analysis of five cpDNA regions (*rbcL*, *atpA*, *matK*, *ndhF*, and *trnL-trnF*) and through micromophological observation of specimens which differs from *H.fudzinoi* by lamina width and exospores. Considering the differences in morphology, geographical range, and genetic distance between these two species, we formally recognized *V.centrochinensis* as an authentic species and proposed a new combination *Haplopteriscentrochinensis* (Ching ex J.F.Cheng) Y.H.Yan, Z.Y.Wei & X.C.Zhang, **comb. nov.** Our findings demonstrate that several taxa in synonyms are missing, and nowadays taxonomy should also include re-evaluation of the past taxonomy.

## Introduction

The question “How many species are there on earth?” is one of the top 125 questions in science, and exploring it is considered equivalent to imagining the number of stars in the sky (Kennedy and Norman 2005). To understand the biodiversity of species, taxonomists should not only explore new species but also re-evaluate the published species’ names that are considered synonymous with older species’ names. According to the stasis of the web of TPL (The Plant List 2013), more than one-third of species names are unclear and approximately one-third of species names are considered synonymous. Unfortunately, once a species name is treated as a synonym, it remains in the pile of synonyms forever. With the development in molecular phylogeny research, DNA barcoding, and detailed taxonomic observations, an increasing number of species names have been re-established from the checklist of synonyms, which ranges from ferns (Shu et al. 2017; Shu et al. 2018; Wei et al. 2018; Wang et al. 2020) to spermatophyte (Luo et al. 2016; Jin et al. 2017; Wang et al. 2018). Consequently, we found that the synonym database could be a new hotspot for biodiversity discovery.

Accurate specimen identification through sequencing of the type specimens or samples from type locality is the key to solving questions regarding taxonomic synonyms. In addition, a clear understanding of the taxonomic status and barcoding database of the species suspected of being independent is required. *Haplopteris* C.Presl is a genus of vittarioid ferns, long treated as a synonym of *Vittaria* Sm. (Kramer 1990; Wu and Ching 1991; Chen et al. 2013). Recently, it has been widely adopted and followed that the Old World *Vittaria* species were transferred to *Haplopteris* (Chen et al. 2013). Because of limited morphology exhibited by members of the *Haplopteris* as well as convergent and parallel evolution of morphological characteristics, the circumscription of species in the genus has been ambiguous. *Vittariacentrochinensis* Ching ex J.F.Cheng, just one of a long sleeping suspicious species in the synonym list of *Haplopteris*, was initially published in “Flora of Jiangxi” (Xu 1993) as a new species and subsequently considered as a synonym of *V.fudzinoi* (Zhang 1999). To date, it has been regarded as a synonym of *Haplopterisfudzinoi* (Makino) E.H.Crane (Zhang and Gilbert 2013; Yan et al. 2016). *Haplopterisfudzinoi* is a species originally described in Japan, and then used to refer to a Chinese fern (Zhang 1999). During our field investigation in Wuyi Mountain (Jiangxi, China), we collected some population samples of *V.centrochinensis* and found some obvious differences between *V.centrochinensis* and *H.fudzinoi*.

In this study, we analyzed morphological characteristics and geographic distribution along with the molecular phylogeny to confirm the identity of *V.centrochinensis* and phylogenetic affinities of this species with *H.fudzinoi*. We hope that this study can provide a paramount example of re-evaluating of synonyms for new insights into biodiversity discovery.

## Materials and methods

### Morphological analyses and geographical distribution

For morphology, the *H.centrochinensis* was compared with similar species by analyzing photographs of type specimens and field photos. The features of rhizome scales were obtained using Nikon SMZ-1500 (Japan). The morphology of spores was observed with a Quanta 250 scanning electron microscope (FEI, USA), and spore size was measured using ImageJ software (Collins 2007). The descriptions of spore ornamentation abided by Wang and Dai (2010) and Ranker et al. (1993). The map of the geographical distribution of two species, namely *H.centrochinensis* and *H.fudzinoi*, was obtained through field investigation and National Specimen Information Infrastructure (**NSII**). The specimens of *H.centrochinensis* in this study were deposited in Shanghai Chenshan Herbarium (**CSH**).

### Phylogenetic analyses

The total genomic DNA was extracted from silica-dried leaves by using a plant total genomic DNA kit (Tiangen, Beijing, China), according to the manufacturer’s instructions. The primers used for amplification and sequencing were shown in Table 1. Sequencing was performed with an ABI 3730xl DNA analyzer (Applied Biosystems, Foster City, CA, USA). The cpDNA sequences of the three samples of *H.centrochinensis* were submitted to GenBank under accession numbers: MW810047–MW810061 (Table 2). In addition, five cpDNA regions of nine species were downloaded from GenBank (Table 3). Of these, the unavailable data (Table 3) were treated as missing data when they were concatenated. The newly generated sequences were assembled and edited using SeqMan (Burland 1999). Subsequently, all sequences were aligned and manually adjusted on MEGA software (v7.0) (Kumar et al. 2016), with default alignment parameters. Alignments of five cpDNA regions were concatenated using PhyloSuite (Zhang et al. 2018). Then the matrix was used to construct phylogenetic trees with maximum likelihood (ML) and MrBayes. Maximum likelihood analysis was conducted using IQ-TREE (Nguyen et al. 2015) integrated in PhyloSuite with standard bootstrap and TVM+F+G4 model. Bayesian analysis was performed using MrBayes (v3.2.6) (Ronquist et al. 2012) with the GTR+F+G4 model. Four Markov chains were run 1,000,000 generations, with the sampling frequency of 100. The standard deviation of split frequencies was set to less than 0.01 to achieve the convergence of the independent runs. A majority-rule consensus tree was constructed to estimate the posterior probabilities (PP); the first 25% of samples were discarded as the burn-in phase.

**Table 1.** List of PCR amplification and sequencing primers used in the study.

Regions	Primer name	Primer sequence (5’-3’)	Reference
*rbcL*	AF	ATGTCACCACAAACGGAGACTAAAGC	Hasebe et al. (1994)
	ESRBCL1361R	TCAGGACTCCACTTACTAGCTTCACG	Schuettpelz and Pryer (2007)
*atpA*	ESATPF412F	GARCARGTTCGACAGCAAGT	Schuettpelz et al. (2006)
	ESTRNR46F	GTATAGGTTCRARTCCTATTGGACG	Schuettpelz et al. (2006)
*matK*	Vt matK1610F*	GCARTCAARCGTTTAATTRGTA	Chen et al. (2013)
	Vt matK rRFQ	TTATTACTGAATTTGGRATCT	Chen et al. (2013)
*ndhF*	Vt ndhF fAYS	GCTTATTCTACHATGTCTCAGYTRGGATATATGG	Kuo et al. (2016)
	Vt trnN 2210R	TCGTGARACGAAAATAGCAGTTTATGG	Kuo et al. (2016)
*trnL-F*	F	ATTTGAACTGGTGACACGAG	Taberlet et al. (1991)
	FernL 1Ir1	GGYAATCCTGAGCCAAATC	Li et al. (2010)

**Table 2.** GenBank accession number of sequences newly generated in this study.

Species	Location	Voucher	GenBank accession number				
			*rbcL*	*atpA*	*matK*	*ndhF*	*trnL-trnF*
*Haplopteriscentrochinensis* **comb. nov.**	Jiangxi, China	YYH15442-1	MW810047	MW810050	MW810053	MW810056	MW810059
*Haplopteriscentrochinensis* **comb. nov.**	Jiangxi, China	YYH15442-2	MW810048	MW810051	MW810054	MW810057	MW810060
*Haplopteriscentrochinensis* **comb. nov.**	Jiangxi, China	YYH15442-3	MW810049	MW810052	MW810055	MW810058	MW810061

**Table 3.** Information on species and GenBank accession numbers used in the study. Dash (-) indicates unavailable data.

Species	Location	Voucher	GenBank accession number				
			*rbcL*	*atpA*	*matK*	*ndhF*	*trnL-trnF*
*Haplopteristaeniophylla* (Copel.) E.H. Crane	Luzon, Philippines	FWL974	–	–	KC812901	KC812935	KC812969
	Nantou, Taiwan, China	Chen1493	–	–	KC812874	KC812908	KC812942
*Haplopterisdoniana* (Mett. ex Hieron.) E.H. Crane	Yunnan, China	Kuo1418	–	–	KC812880	KC812914	KC812948
	Tamdao, Vietnam	Kuo1801	–	–	KC812905	KC812939	KC812973
*Haplopterisfudzinoi* (Makino) E.H. Crane	Sichuan, China	Kuo2225	KX165003	KX165201	KC812895	KC812929	KC812963
*Haplopterislinearifolia* (Ching) X.C. Zhang	Yunnan, China	Liu9457	KX165012	KX165209	KC812899	KC812933	KC812967
*Haplopterismediosora* (Hayata) X.C. Zhang	Nantou, Taiwan	Chen1492	KX165015	KX165211	KC812875	KC812909	KC812943
*Haplopterisamboinensis* (Fée) X.C. Zhang	Hainan, China	Kuo1715	–	–	KC82879	KC812913	KC812947
*Haplopterisflexuosa* (Fée) E. H. Crane	Yunnan, China	Kuo1142	–	–	KC812881	KC812915	KC812949
*Antrophyumparvulum* Blume	Nantou, Taiwan, China	Chen1495	–	–	KC812877	KC812911	KC812945
*Antrophyumsessilifolium* (Cav.) Spreng	Taitung, Taiwan, China	Chen1502	KX164974	KX165181	KC812876	KC812910	KC812944

## Results

### Morphological comparisons and geographical distribution

**Figure 1.**
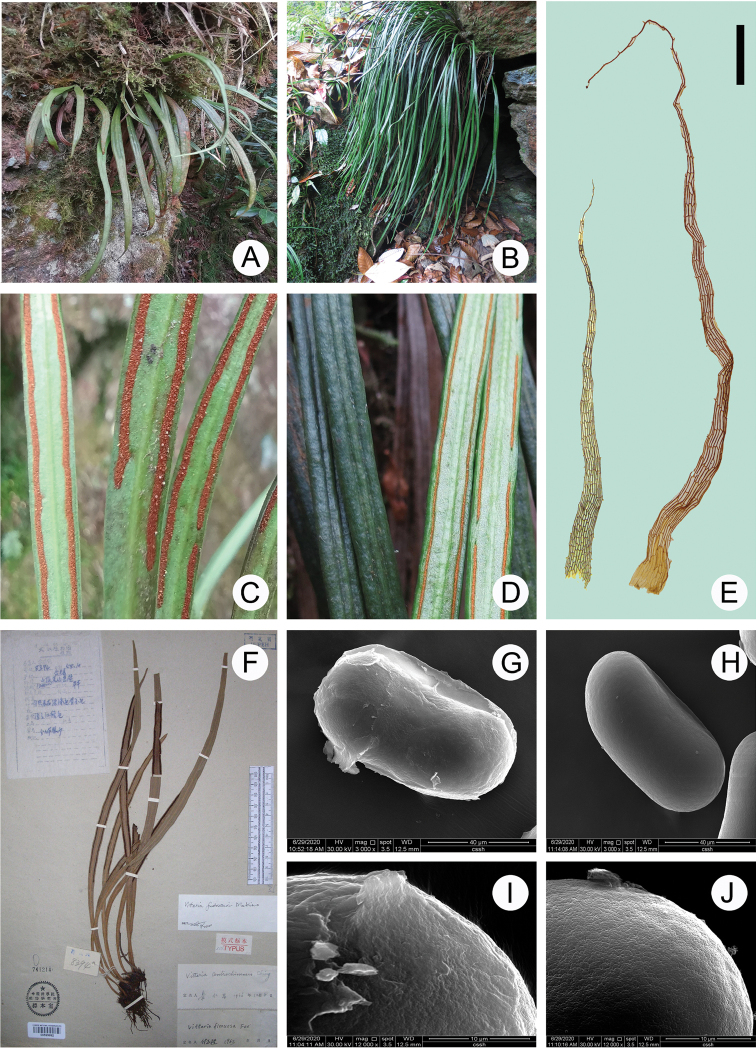
Morphological observations in *H.centrochinensis* YYH15442 (**A, C, F, G, I**) and *H.fudzinoi* SG1654 (**B, D, H, J**) **A** habitat **C** sorus position and flat lamina **F** type specimen (provided by National Plant Specimen Resource Center, http://www.cvh.ac.cn); and **G, I** spore and ornamentation in *H.centrochinensis* YYH15442 **B** habitat (taken by Hong-Jin Wei) **D** sorus position and flat lamina (taken by Hong-Jin Wei) **H, J** spore and ornamentation in *H.fudzinoi* SG1654 **E** rhizome scale, left: *H.fudzinoi*, right: *H.centrochinensis*.

The morphological and micromorphological characters of *H.centrochinensis* and *H.fudzinoi* are presented in Figure 1 and Table 4. The lamina of *H.centrochinensis* was shorter and wider than that of *H.fudzinoi* (Fig. 1A, B, F); in *H.fudzinoi* costa it was raised adaxially with two prominent long grooves beside the costa on adaxial surface (Fig. 1D). The rhizome scales were yellow-brown, margin denticulate, linear-lanceolate, and clathrate (Fig. 1E left); scale margins of *H.centrochinensis* were toothed and those of *H.fudzinoi* were subentire to minutely denticulate at lower margin and upper part, respectively. The scales length of *H.centrochinensis* were visibly longer than that of *H.fudzinoi* (Fig. 1E). Spores were monolete for both the species. Spore ornamentation observed in *H.centrochinensis* was scabrate and rugate (Fig. 1G, I), whereas it was laevigate or inconspicuous-granulate in *H.fudzinoi* (Fig. 1H, J). Additionally, sori position was distinct between the two species; the soral line in *H.fudzinoi* was located close to the edge of lamina and immersed in groove (Fig. 1D), whereas it was immersed between the frond costa and margin in *H.centrochinensis* (Fig. 1C). The geographical distribution for these two species was obtained on the basis of the information of the specimens. The result indicated that most distributions are shared by *H.centrochinensis* and *H.fudzinoi* (Fig. 3).

**Table 4.** Morphological comparisons between *H.centrochinensis* and *H.fudzinoi*.

**Features**	***H.centrochinensis***	***H.fudzinoi***
Lamina width	10–15 mm	8–10 mm
Lamina margin	Flat	Reflexed
Adaxial costa	Slightly raised	Greatly raised
Abaxial costa	Carinated	Sharp carinate
Rhizome scale	Long, margin toothed	Short, lower margin subentire, upper part minutely denticulate
Exospores	Scabrate	Psilate
Sorus position	Between the frond costa and margin	Close to the lamina edge

### *Haplopteriscentrochinensis* is an independent species according to molecular data obtained using phylogenetic analysis

**Figure 2.**
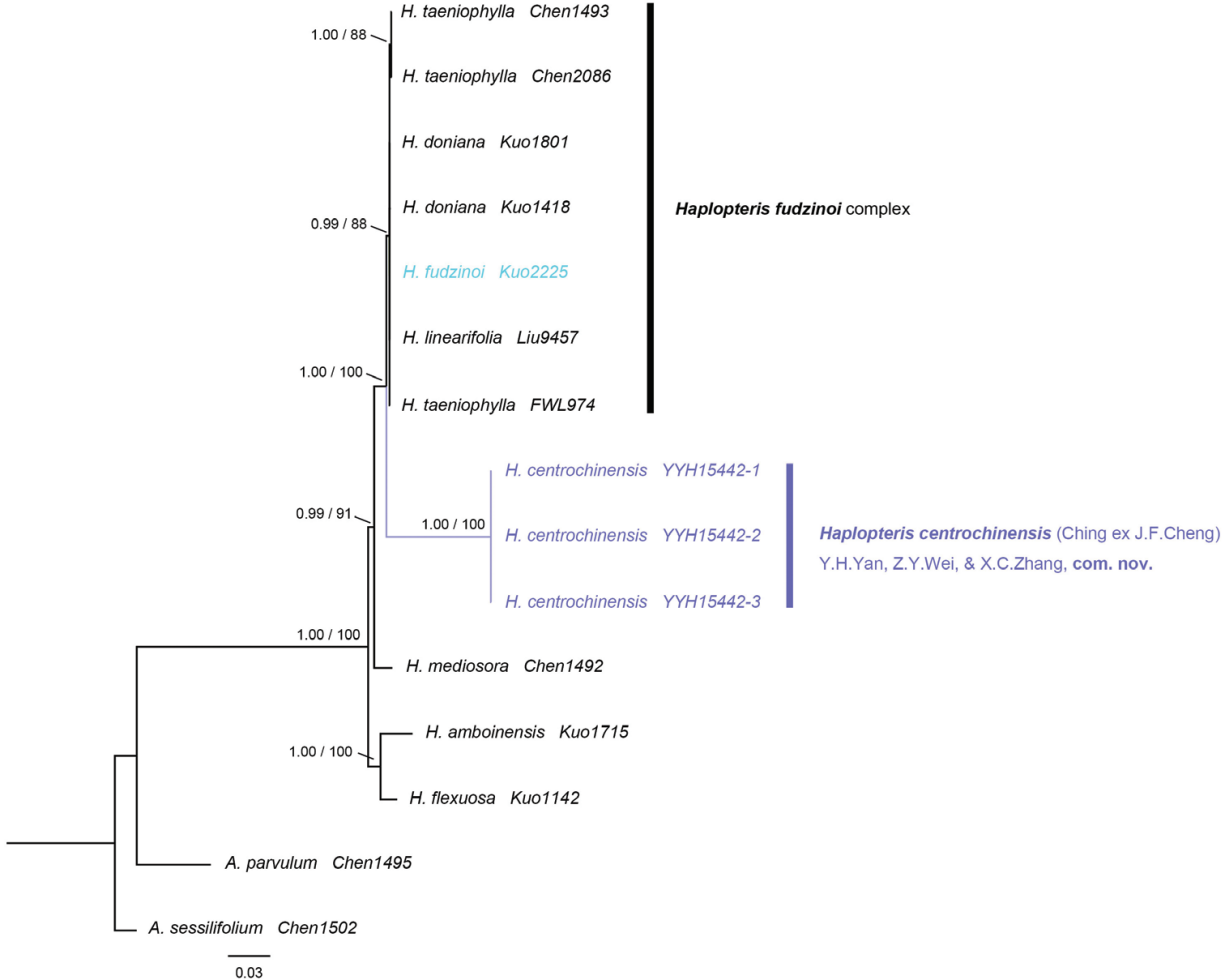
Majority consensus tree derived from Bayesian tree based on 5 cpDNA loci (*rbcL*, *atpA*, *matK*, *ndhF*, and *trnL-F*). Numbers above the branches are support values in the order of PP_BI_/BS_ML_.

The two phylogenetic analyses (BI, ML) recovered congruent topologies, with *Antrophyumparvulum* and *Antrophyumsessilifolium* as outgroups (Fig. 2). The results revealed strong support for the monophyly of *H.centrochinensis* (PP = 1.0, BS = 100) (Fig. 2), and it was strongly supported as a sister to another four species (i.e., *H.fudzinoi*, *H.doniana*, *H.taeniophylla*, and *H.linearifolia*) of *Haplopteris* (PP = 1.0, BS = 100) (Fig. 2). The genetic distance between the *H.centrochinensis* and these four *Haplopteris* species ranged from 0.073 to 0.120, and the intraspecific distances of these four species were 0 and 0.001 (Table 5).

**Table 5.** Genetic distance between eight individuals of five *Haplopteris* species.

	**1**	**2**	**3**	**4**	**5**	**6**	**7**
2	0.073*						
3	0.120*	0.001					
4	0.120*	0	0				
5	0.120*	0.001	0.001	0.001			
6	0.120*	0.001	0.001	0.001	0		
7	0.120*	0	0	0	0.001	0.001	
8	0.073*	0	0	0.000	0.001	0.001	0

Note: 1 = *H.centrochinensis* (YYH15442); 2 = *H.fudzinoi* (Kuo2225); 3 = *H.donoana* (Kuo 1418); 4 = *H.doniana* (Kuo1801); 5 = *H.taeniophylla* (Chen 2086); 6 = *H.taeniophylla* (Chen 1493); 7 = *H.taeniophylla* (FWL 974); 8 = *H.linearifolia* (Liu 9457); Genetic distances of *H.centrochinensis* from others species are shown with *.

**Figure 3.**
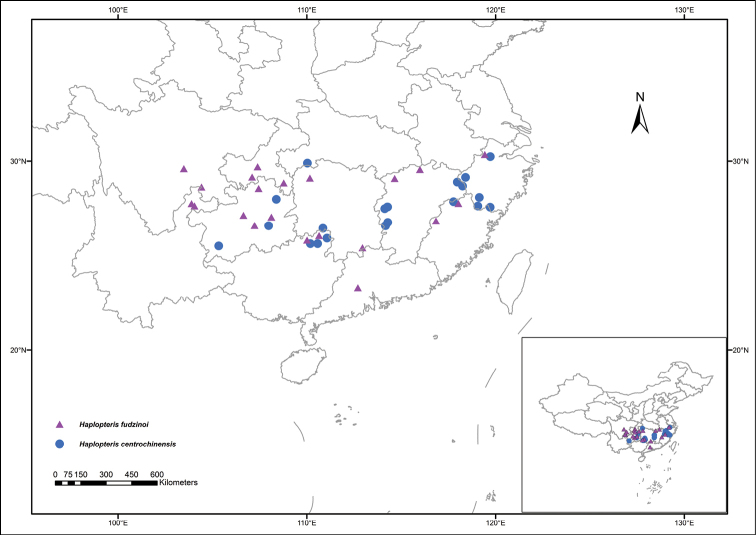
Geographic distribution of *H.centrochinensis* and *H.fudzinoi* in China. The dataset is provided by the National Specimen Information Infrastructure (http://www.nsii.org.cn).

## Discussion

### Re-evaluation of synonyms for new insights into biodiversity discovery

Synonym is the first concern in the estimation of the total number of species in one taxon, and only after its resolution can one ask the next question regarding how many additional species there are in the taxon (Joppa and Pimm 2011). Surprisingly, nearly two-thirds of the plant names are synonymous or recorded as unresolved in *TPL* (2013), which consists of 26,000 additional synonyms that were not listed in its earlier version (v.1.0). The increase in the number of newly discovered species has been consistent in line with the use of molecular evidence; however, information on synonyms is meager. For instance, International Plant Names Index (IPNI 2020) provides information on nomen novum, combination nova, and taxa nova, but it provides no information on new species resurrected from the established synonyms. Although many species of ferns (Liu et al. 2013; Morigengaowa et al. 2018; Shu et al. 2017; Shu et al. 2018; Sigel et al. 2014; Wang et al. 2020; Wei et al. 2018) and seed plants (Tkach et al. 2015; Hu et al. 2015) have been reinstated as independent species, this type of study has been rarely reported. The extent of biodiversity hidden in taxonomic literature is an interesting question to explore. To understand the biodiversity of our planet and for efficient conservation of valuable natural resources, the prime objective of taxonomists should be to correctly identify our planet biodiversity by using modern taxonomic facilities.

**Figure 4.**
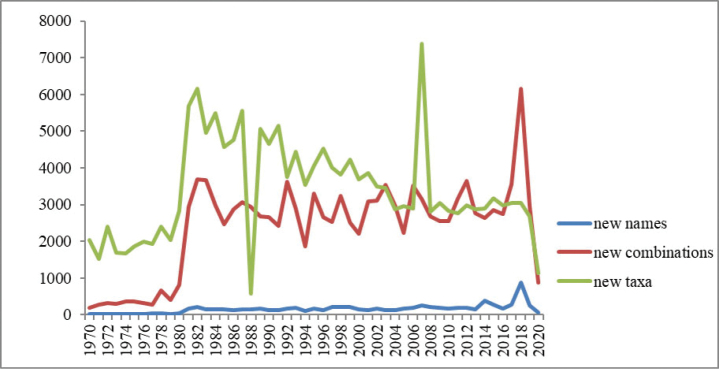
Trends in the number of new names, new combinations, and new taxa published over 50 years (1970–2020).

Various taxa, especially widely distributed ones, still require a comprehensive systematic treatment that also involves evaluating their nomenclature. Then, if cryptic taxa or misunderstood species have to be segregated, naming these taxa needs first to be evaluated against synonymy as potential sources of the needed name, otherwise a new name needs to be proposed. However, the number of taxonomists has significantly declined (Haas and Hauser 2005), and young taxonomists do not pay enough attention either to the evaluation of synonymous names already listed in the taxonomy or to the assessment of thousands of different species names and their type specimens. Therefore, the number of species on earth remains uncertain. Fortunately, technological advancement has considerably affected taxonomy. According to the IPNI data, two obvious turning points in taxonomy have affected the trends in new combinations and new taxa (Fig. 4), and those are related to the development of electron microscopy and molecular phylogeny in the 1970s and the 1990s, respectively (Endess et al. 2000). Although the new taxa have been displaying a steady or even a downward trend, the new combination is expected to display an upward trend in future, with the application of molecular biology in taxonomy (Fig. 4). Unsurprisingly, new combinations will continue to occur for a long time because of the abundance of listed synonyms and suspected species names, which are equivalent to the new species in the wild. Thus, synonyms and suspected species will serve as the new biological diversity hotspot for the exploration of new unknown species.

### Integrative taxonomy contributes to clarifying species delimitation

The reason for numerous synonyms existing only in books may be the lack of sufficient morphological judgments made in the past. In the present study, the phylogeny (Fig. 2) based on the 5-locus dataset revealed strong support for the monophyly of *H.centrochinensis* (PP = 1.0, BS = 100) (Fig. 2) and strong support for *H.centrochinensis* as sister to *H.fudzinoi* (PP = 1.0, BS = 100) (Fig. 2). Although our result was different from that of a study by Zhang and Gilbert (2013) that indicated distinction between the two species, no further research was performed merely because of limited conditions at that time. Moreover, genetic distance in line with the K2P model showed an obvious hereditary difference between the *H.centrochinensis* and another three *Haplopteris* species (Table 5). For morphological comparisons, several traits were observed. Of these, the most unambiguous differences between the two species (*H.centrochinensis* and *H.fudzinoi*) were the wider lamina, longer rhizome scale, and shorter stipe in *H.centrochinensis*. In addition, *H.fudzinoi* costa was raised adaxially with two prominent long grooves besides the costa on adaxial surface. Furthermore, the scabrate and rugate ornamentation of spores observed in *H.centrochinensis* was found to be laevigate or inconspicuous-granulate in *H.fudzinoi*. To sum up, monophyletic clade, long genetic distance, stable morphological differentiation, and independent geographical distribution form the basis of establishing *H.centrochinensis* as a divergent species or an independent species, and therefore, it should not be considered synonymous with *H.fudzinoi*.

Here, we proposed a new combination *H.centrochinensis* (Ching ex J.F.Cheng) Y.H.Yan, Z.Y.Wei & X.C.Zhang, comb. nov. The taxonomic treatment of *H.centrochinensis* is as follows.

### Taxonomic treatment

#### Haplopteris centrochinensis

Taxon classificationPlantaePolypodialesPteridaceae

(Ching ex J.F. Cheng) Y.H.Yan, Z.Y.Wei & X.C.Zhang comb. nov.

1CB5E3B0-EABD-5990-82DB-4AAA2B619564

urn:lsid:ipni.org:names:77217251-1

Vittaria centrochinensis Ching ex J.F.Cheng: Fl. Jiangxi 1: 365. 1993. Basionym.
Vittaria taeniophylla sensu F.Zhang, non Copel.: Fl. Zhejiang 1: 111. 1993. p.p.
Vittaria fudzinoi sensu X.C.Zhang, non Makino: Fl. Rep. Poup. Sin. 3(2):20.1999. p.p.
Haplopteris fudzinoi sensu Zhang & Gilbert, non (Makino) E. H. Crane: Fl. China 2(3): 254.2013. p.p.

##### Type.

China. Hubei Province, Enshi Tujia and Miao Autonomous Prefecture, Hefeng District, elev. 1200 m, October 1958, Hong-Jun Li, 8394 (holotype, PE!; isotypes, IBSC!, NAS!).

##### Additional specimens examined.

**Guangxi Province**: Damiaoshan District, 26 July 1958, Shao-Qing Chen, 15853 (IBSC); Quanzhou District, April 27, 2013, Quanzhou census team, 450324130427042LY (GXMG). **Guizhou Province**: Kaili City, census team, 3592 (CNBG); Xingren District, 9 August 1960, census team, 7872 (CNBG); Yinjiang, December 26, 1930, Y. Tsiang, 7867 (CNBG). **Hunan Province**: Shaoyang City, Dongkou District, 24 May 1983, Ze-Yong Yang, 166 (IBSC); Xinning District, September 9, 1984, Anonymous, 394 (PE). **Jiangxi Province**: Shangrao City, Yanshan District, Wuyi Mountain National Nature Reserve, 1729 m, October 7, 2019, Yue-Hong Yan, Zuo-Ying. Wei, Quan Yuan, YYH15442 (NOCC); Shangrao City, Yushan District, Sanqingshan, July 27, 1991, Sheng-Xiu Xu, 91018 (JXU); Jinggangshan City, Jinggangshan, April 13, 1983, Sheng-Xiu Xu, 83422 (JXU); Jinggangshan City, Jinggangshan, February 1982, 8210118 (JXU); Jinggangshan City, Jinggangshan, November 4, 1982, 8220349 (JXU); Jinggangshan City, Jinggangshan, July 2, 1973, Jing -Fu Cheng, 730433 (JXU); Shangrao City, Yushan District, Huaiyushan, July 1970, 0028466 (PEY); Pingxiang City, Luxi District, March 24, 2014, Gong-Xi, Chen and Dai-Gui Zhang, LXP-06-1246, LXP-06-1251, LXP-06-1201 (SYS). **Zhejiang Province**: Quzhou City, Kaihua District, September 1, 2019, She-Lang Jin, Hong-Yu Wei, Jiao Zhang, JSL5850 (CSH); Longquan City, September 27, 1963, Shao-Guang Zhang, 4453 (CNBG); Linan City, May 25, 1958, Anonymous, 28714; Qingyuan District, Pei-Xi Qiu, 3935 (PE); Taishun District, July 17, 1960, Anonymous, 8576 (CNBG).

##### Note.

*Vittariacentrochinensis* Ching ex J.F.Cheng was initially published in “Flora of Jiangxi” as a new species found in two distributed provinces (i.e., Jiangxi and Hubei). The type locality is situated in the Hefeng District from which a single specimen was cited. Additional specimens were cited from Jiangxi Province.

## Supplementary Material

XML Treatment for Haplopteris centrochinensis
